# A Weighted Genomic Relationship Matrix Based on Fixation Index (F_ST_) Prioritized SNPs for Genomic Selection

**DOI:** 10.3390/genes10110922

**Published:** 2019-11-12

**Authors:** Ling-Yun Chang, Sajjad Toghiani, El Hamidi Hay, Samuel E. Aggrey, Romdhane Rekaya

**Affiliations:** 1 Department of Animal and Dairy Science, University of Georgia, Athens, GA 30602, USA; Sajjad.Toghiani@ARS.USDA.GOV (S.T.); rrekaya@uga.edu (R.R.); 2 ABS Global, Inc., DeForest, WI 53532, USA; 3 USDA Agricultural Research Service, Fort Keogh Livestock and Range Research Laboratory, Miles City, MT 59301, USA; ElHamidi.Hay@ARS.USDA.GOV; 4 Department of Poultry Science, University of Georgia, Athens, GA 30602, USA; saggrey@uga.edu; 5 Institute of Bioinformatics, University of Georgia, Athens, GA 30602, USA

**Keywords:** high density, sequence data, genomic selection, accuracy

## Abstract

A dramatic increase in the density of marker panels has been expected to increase the accuracy of genomic selection (GS), unfortunately, little to no improvement has been observed. By including all variants in the association model, the dimensionality of the problem should be dramatically increased, and it could undoubtedly reduce the statistical power. Using all Single nucleotide polymorphisms (SNPs) to compute the genomic relationship matrix (**G**) does not necessarily increase accuracy as the additive relationships can be accurately estimated using a much smaller number of markers. Due to these limitations, variant prioritization has become a necessity to improve accuracy. The fixation index (F_ST_) as a measure of population differentiation has been used to identify genome segments and variants under selection pressure. Using prioritized variants has increased the accuracy of GS. Additionally, F_ST_ can be used to weight the relative contribution of prioritized SNPs in computing **G**. In this study, relative weights based on F_ST_ scores were developed and incorporated into the calculation of **G** and their impact on the estimation of variance components and accuracy was assessed. The results showed that prioritizing SNPs based on their F_ST_ scores resulted in an increase in the genetic similarity between training and validation animals and improved the accuracy of GS by more than 5%.

## 1. Introduction

Recent advances in high-throughput genotyping and sequencing techniques have led to the generation of dense marker panels and facilitated the genotyping of large numbers of individuals. Because of the availability of these cost-effective genotyping technologies and the increase in sequencing speed, large-scale genotyping for single-nucleotide polymorphisms (SNP) has become more affordable and accessible. Genomic data provide an unprecedented opportunity to dissect the genetic basis of complex traits and identify relevant functional associations. 

From an animal breeding perspective, the use of genomic information results in a substantial reduction in generation interval and an increase in the accuracy of predicted breeding values, leading undoubtedly to an improvement in the genetic response [1,2,3,4,5,6]. Genomic selection (GS) is often carried out using multiple regression or mixed linear models [7,8,9,10,11,12]. For both methods, the density of the SNP marker panel and the linkage disequilibrium (LD) structure between markers and quantitative trait loci (QTL) have a great impact on accuracy. Regression-based methods directly model the association between the phenotypes and all or a subset of the genotyped variants. Thus, their problems stem mainly from the high dimensionality of the parameter space. As the effect of a QTL (often small for complex traits) is distributed in a nontrivial manner between all markers that are in LD with the causal mutation, there is little statistical power to accurately estimate its effect. Traditionally, SNP filtering is conducted based on certain statistical criteria such as p-values for single-marker analyses or quality-of-fit and model determination for Bayesian procedures such as BayesB [13] and BayesR [14]. The latter has shown some superiority for certain traits in the presence of low- and moderate-density marker panels as compared with models that include all markers, however, they still suffer, although to a lesser degree, from high false positives, multiple testing problems, high LD, and small SNP effects, which have hampered at different degrees the efficiency of these methods [15,16,17]. Although these factors are likely to affect the prioritization of relevant variants, they have limited to no effects on prediction. An increase in SNP marker density, after a certain threshold, seems to not affect the quality of the estimated observed relationship matrix (**G**) and thus the performance of mixed linear model-based approaches. There was no difference in accuracy between the 777K SNP and the 54K SNP panels [18]. This is because the quality of **G** when either 777K or 54K SNP panel were used was not that different. Due to these limitations, prioritization of variants to be included in the association model or to compute the genomic relationship matrix has become a necessity. Commercial livestock species are under heavy artificial selection. The effects of such selection on the genome can be traced through the changes in allele frequencies. The fixation index (F_ST_) measures the rate of fixation through the increase in homozygosity and it has become an important tool to study population structure in humans, animals and plants. Chang et al. [19] proposed utilizing the F_ST_ which measures the allele differentiation among subpopulations to identify segments of the genome under selection pressure. There was an increased genomic similarity and improvement in the accuracy of genomic selection when the F_ST_ scores were used to prioritize SNP markers in high-density panels as compared with using BayesB [20] and BayesC [21] approaches. Furthermore, they showed that the genomic relationship matrix and the accuracy could be improved using prioritized SNPs based on the F_ST_ scores. 

Genomic best linear unbiased prediction (GBLUP) assumes equal weight for all SNPs [22,23]. Sun et al. [24] developed a two-step method for calculating weights in weighted GBLUP (WGBLUP). If weights are known, WGBLUP calculates genomic estimated breeding values (GEBVs) similar to the Bayesian method using the same weight. This method is effective for distinguishing major QTL, however, the accuracy of the GEBVs is reduced since it shrinks small SNP effects to zero. To achieve the highest accuracy, the weight formula needs to be modified to avoid having SNPs with no effect. The use of a genomic relationship matrix that weights marker’s contribution can improve prediction accuracy, but the improvement is trait and population specific due to differences in genetic architecture. Weighted single-step GBLUP (WssGBLUP) have been developed for estimating weights within single-step GBLUP (ssGBLUP) process [25,26]. Wang et al. [26] and Fragomeni et al. [27] evaluated the performance of the WssGBLUP approach using simulation data. Two iterations of weights were calculated from the variance explained by each SNP. Their results showed that weighting SNP could be effective in improving the accuracy of GEBV prediction and in the estimation of marker effects. 

With all these methods, the challenge was to determine how to derive the optimum set of weights to compute the genomic relationship matrix. In this study, the F_ST_ scores-based prioritization method developed by our group (Toghiani et al. [28] and Chang et al. [19]) has been be expanded to derive the needed weights to compute **G**. The specific objectives of this study were to derive F_ST_ scores-based relative weights for SNPs included in the computation of **G** and to assess the impact of different strategies on the estimation of variance components and the accuracy of genomic selection.

## 2. Materials and Methods

### 2.1. Data Simulation

The QMSim simulation software [29] was used to simulate the genomic and phenotypic data. A randomly mated historical population was generated to initialize LD and to establish mutation-drift equilibrium and was used as a base to create a population with average LD between adjacent markers of 0.3. Three hundred historical generations were simulated based on random mating of an initial 8,000 animals, followed by an additional 5, 10, and 20 generations with population ranging between 12,000 and 17,000 animals. The base population (G_0_) was founded by 1000 males and 15,000 females randomly selected from the historical generation. A trait with heritability equal to 0.30 was simulated and all genetic variation was assumed to be due to the simulated QTL. The mating system was at random throughout up to generation G_0_. We also simulated an additional 15,000 animals for each of 7 additional generations (G_1_–G_7_). The parents were sampled on their estimated breeding values (EBVs), with a replacement rate of 50 and 20% for males and females, respectively. We assumed one progeny per mating and a sex ratio of 50%. Each simulation scenario was replicated 5 times. The average of the effective population size was equal to 323. Data from generation 6 (G_6_) was used as a training population and that of generation 7 (G_7_) was used to evaluate (validation population) the proposed method. All animals in the training and validation populations were genotyped with 400,000 SNP markers simulated to be uniformly distributed along 10 chromosomes of 100 cM in length each to approximate about 1.2 million SNP markers in the bovine genome. We sampled 200 biallelic QTL from a Gamma distribution with shape and scale parameters equal to 0.4 and 0.15 respectively. We did not allow any overlap between the SNP markers and QTL. 

Additionally, QTL were assumed not to be genotyped. The residual variance was scaled accordantly in each scenario of selected SNPs such that the heritability and phenotypic variance were constant at the values of 0.3 and 1, respectively. Trait phenotypes were generated as the sum of an overall mean, the random additive effects of QTL and their associated genotypes and the residual terms. The latter were sampled from a normal distribution with zero mean and variance-covariance matrices **I**σ_e_^2^ where σ_e_^2^ is the residual variance.

### 2.2. SNPs Prioritization Based on F_ST_ Scores

Briefly, divergence between populations and subpopulations is often due to differential selection pressure. Wright’s fixation indexes (F_ST_) have been used to measure the level of genetic differentiation between populations based on change in allele frequencies. The F_ST_ scores were calculated following Nei [30] and Chang et al. [19]. Specifically, the trait phenotypes for animals in generation 6 (G_6_) were divided into three sub-populations based on the 5% and 95% quantiles (below the 5% quantile (S_1_), between 5% and 95% quantiles (S_0_), and above the 95% quantile (S_2_)). Genotypes of individuals (1500) in sub-populations S_1_ and S_2_ were used to calculate the F_ST_ scores. For each locus, the global F_ST_ estimator was defined as:

(1) $$F_{ST}=\frac{H_{T}-H_{S}}{H_{T}}\;\mathrm{with}\;H_{T}=2\;\times \;p\;\times \;q,\;H_{S}=\frac{H_{S1}*n_{s1}+H_{S2}*n_{s2}}{n_{s1}+n_{s2}},\;\mathrm{and}\;H_{Si}=2\;\times \;p_{Si}\;\times \;q_{Si}$$

where *p_Si_* and *q_Si_* are the allele frequencies in subpopulation *i*, *n_S_*_1_ and *n_S_*_2_ are the number of individuals of the subpopulations *S*_1_ and *S*_2_, *H_S_* is the average heterozygosity of subpopulations, and *H_T_* is the heterozygosity based on the total population.

### 2.3. Prioritized SNPs and Genomic Relationships

Several methods have been proposed to calculate the genomic relationships [31,32,33,34,35]. In animal breeding applications, the genomic relationship matrix (**G**) is often calculated using the method proposed by VanRaden [32]. It basically measures the similarity of marker genotypes between two individuals at a large number of loci independent of their mode of inheritance. Estimating observed additive relationships using identity by state provides a better estimate than using pedigree information, but still suffers from several problems including nonzero estimates of realized relationship between two individuals that are not related by ancestry as it was shown by [36,37,38], negative off-diagonal elements, and the inevitable noise associated with these estimates. Furthermore, several studies [4,18,39] have shown that little to no improvement in **G** were observed with an increase in the number of SNPs used for its calculation. Current methods used to calculate **G**, generally, give the same weight to all the markers, and thus could not guarantee the optimality of genetic similarity between individuals at the QTL. For that purpose, contributions of the SNPs used to compute **G** have to be weighted according to their importance on the phenotype (strength of association with the phenotype). To maximize the functional genomic similarity between individuals, the SNPs have to be prioritized based on their ability to increase genetic or phenotypic similarity between individuals. Conversely, individuals with different genetic values or phenotypes are likely to have much lower genomic similarity at QTL than the expected or observed additive relationships. It is worth mentioning that F_ST_ is only a measure of population differentiation and an increase in functional similarity is achieved through an increase of the relative weight of prioritized markers.

The challenge in maximizing the genomic similarities is finding the relative weights for the SNPs used in the calculation of **G**. In this study, F_ST_ scores were used to prioritize and to assign relative weights to the SNP markers. The top 20K SNPs based on their F_ST_ scores were used either alone or with the remaining 380K SNPs to compute **G** with or without weighting. When only the top 20K SNPs were used to compute **G**, the following two scenarios were considered: 1) equal weights for all SNPs or 2) weights proportional to each SNP F_ST_ score. When all 400K SNP markers were used, the different weighting scenarios evaluated are presented in Table 1.

The relative weights were calculated using the following equation:

(2) $$w_{i}=\;\frac{F_{ST_{i}}}{\sum_{j=1}^{N}F_{ST_{j}}}\;\times \;N$$

where $w_{i}$ is the relative weight for SNP *i*, $\;Fst_{i}$ is the F_ST_ score for SNP *i* and *N* is the total number of SNPs (400K or 20K).

### 2.4. Data Analysis

For all scenarios, 10,000 and 5000 animals were randomly selected from G_6_ and G_7_, respectively. For each scenario, the genomic relationship matrix was computed with the appropriate number of markers and the weighting factors and the analysis was carried out using the following mixed linear model:

(3) y=Xb+Zu+ e

where y is a *N* × 1 column vector of phenotypes, *X* is a *N* × *p* known incidence matrix of the *p* predictor variables, *b* is a *p* × 1 column vector of fixed effects regression coefficients, *Z* is a *N* × *q* known incidence matrices with the appropriate dimensions for the *q* random effects, *u* is a *q* × 1 column vector of genomic breeding values, and *e* is a *N* × 1 column vector of random residuals. Additionally, it was assumed that $u\sim N\left(0,\;G\sigma_{u}^{2}\right)$, with $\sigma_{u}^{2}$ being the genetic variance.

The AIREMLF90 program [40] was used to estimate variance components and to predict the genomic breeding values for the different scenarios. Accuracy of genomic evaluation was defined as the correlation between true and estimated breeding values in the validation population. Each simulation scenario was replicated 5 times.

## 3. Results

Table 1 presents the estimates of the variance components and heritability and their associated standard deviations for the different scenarios when all 400K SNP markers were used to compute the genomic relationship matrix. In general, the percentage of genetic variance recovered increased with a decrease of the percentage weight assigned to the prioritized top 20K SNPs reaching a maximum when the top SNPs (based on F_ST_ scores) accounted for 25% or less of the weights used to compute **G**. In all cases, the genetic variance was underestimated when no weights were used (scenario 7 in Table 1). Similarly, only two-thirds of the genetic variance were recovered when zero weights were assigned to the 380K nonprioritized SNPs (scenario 1 in Table 1). 

Table 2 presents the distribution of off-diagonal elements of **G** for different weighting scenarios. In fact, the portion of genomic relationships between training and validation individuals exceeding 0.03 was 5.24% when all 400K SNPs were used with equal weight. The same portion was 5.59%, 7.22%, 11.13%, 14.38%, and 16.78% when the relative weight assigned to the top 20K prioritized SNPs in the calculation of **G** was 25%, 50%, 75%, 90% and 100%, respectively. When only the top 20K prioritized SNPs were used to compute G, weighting the contribution of each marker by its F_ST_ score resulted in an increase in the off-diagonal elements exceeding 0.03 (Table 3). The increase in the percentage of off-diagonal elements exceeding 0.03 is an indicator of increased similarity between the training and validation datasets and could lead to increase in accuracy.

**Figure 1:**
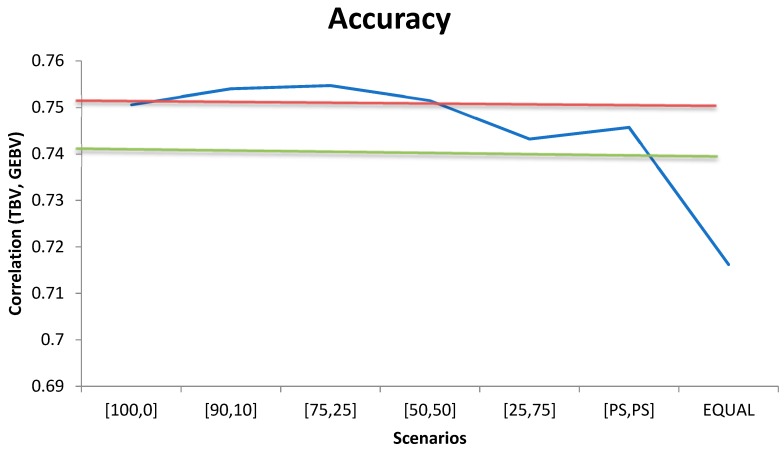
Accuracy of genomic prediction for different weighting scenarios for the contribution of the 20K prioritized SNPs and the remaining 380K markers (x,y). Horizontal lines indicate the accuracy using only the top 20K SNPs with (red) or without (green) weights SNPs.

When the same weight ($w_{i}=1)$ was used for all 400K SNP markers to compute **G**, the accuracy of genomic prediction (correlation between true and predicted BVs) was 0.690 (Figure 1) and it increased to 0.718 when all SNPs in the panel were weighted by their relative F_ST_ score. When the relative weight of the top 20K prioritized SNPs in the calculation of **G** increased, higher accuracy was achieved. In fact, accuracy increased by 4.3%, 5.2%, 5.4%, 5.3%, and 5.2% as compared with the scenario where all markers had the same weight ($w_{i}=1)$ when the relative weight assigned to the top 20K prioritized SNPs in the calculation of **G** was 25%, 50%, 75%, 90% and 100%, respectively (Figure 1). 

A comparison between the different weighting scenarios for the contribution of the 20K prioritized SNPs and the remaining 380K markers showed a superiority for scenarios one to six as compared with scenario seven (equal weights) in terms of quality-of-fit of the model (Table 4). In fact, the −2Log likelihood ranged from 26,397.30 to 26,746.90 for scenarios one to six with the best fit being for the third and fourth scenarios. When the same weights were assigned to all 400K SNP (scenario seven), the −2Log likelihood was 27,378.30. Similar behavior was observed for the estimated residual variance (Table 4). Regression of the estimated breeding values on the true ones showed a systematic under estimation for all seven scenarios, although the bias was slightly smaller for scenarios one to six (Table 4). 

## 4. Discussion

We showed that only a portion of the genetic variance was recovered for the different scenarios. The inability to recover all the genetic variance is due to the large number of QTL with very small effects. In fact, 55% of QTL have a true effect smaller than one-tenth of one percent of the genetic variance and an additional 20% of QTL have an effect smaller than 0.5% of the total genetic variance. These small effect QTL are hard to track effectively when the LD is moderate to low. Across the different scenarios, there is an underestimation trend of the residual variance, although it does not seem to be any systematic bias. Heritability was clearly underestimated when the majority of the weight (≥90%) was allocated to the prioritized top 20K SNPs (scenarios one and two in Table 2). In fact, for those scenarios, estimates of the heritability are likely to be biased. For the remaining scenarios, although there is a general trend of an underestimation of the heritability, estimates are not likely to be biased. When only the unweighted top 20K prioritized SNPs were used to compute **G**, the genetic and residual variances were very similar to the estimates obtained for scenario one in Table 2. 

Intrinsically, the contribution of a SNP marker to the estimation of **G** is weighted by its minor allele frequency (MAF), thus favoring markers with low MAF. However, it is not weighed by the size of the marker effect. Consequently, after a certain number of SNP markers are included in the computation of **G**, little to no improvement is expected. Chang et al. [19] showed that the limited change in **G** with additional markers could be an indicator of the sufficiency of available SNPs in estimating the realized relationships. However, such sufficiency is not a guarantee of the optimality of such matrix for the implementation of association and genome selection analyses. In fact, as the number of randomly selected SNPs increased from 40K to 400K, the matrix **G** inched closer to the expected additive relationship matrix (**A**). Furthermore, they showed that a genomic relationship matrix computed based on a selected subset on 20K markers was markedly different from **A**. In this study, we further prove that within those selected 20K SNPs additional improvements could be achieved through appropriate weighting of the contribution of these SNPs in the calculation of **G**.

Weighting all markers with their relative F_ST_ scores resulted in a 4.3% increase in accuracy as compared with the same weight scenario ($w_{i}=1)$. Using only the prioritized 20K SNPs with or without weights resulted in a 5.2% and 3.5% increase in accuracy as compared with the same weight scenario. As the density of the marker panel increases, using all SNPs to compute **G** is not the best option. 

These results clearly indicate that additive relationships between individuals could be accurately estimated with a reasonably small number of well distributed SNP markers, however, that does not mean that the accuracy of genomic selection cannot be improved using high-density marker panels or even sequence data. To achieve that goal, the genomic matrix has to evolve from a measure of additive relationships to an optimum measure of genetic similarity at QTL between individuals. The F_ST_ scores seem to be an efficient prioritization tool to achieve such a goal, however, it should be noted that F_ST_ scores are only measures of fixation index. A combination of metrics of fixation index and index of genetic differentiation could lead to better representation of population partitioning [41] and could enhance the prioritization and weighting of SNP markers.

## 5. Conclusions

The dramatic increase in the number of identified common and rare variants due to advances in NGS was expected to significantly increase the accuracy of GWAS and GS. Unfortunately, little to no improvement in accuracy was observed using NGS or high-density marker data. In spite of the repeated argument that all needed information is already captured by the available marker panels, the results of this study clearly show that the lack of improvement in accuracy is due to the limitations of the methods used rather than the limited additional information in the high-density and sequence data. Prioritizing SNP markers based on their F_ST_ scores and using the latter to compute relative weights has increased the genetic similarity between training and validation animals. Furthermore, it resulted in more than 5% improvement in accuracy. These results clearly indicate that additive relationships between individuals could be accurately estimated with a reasonably small number of well distributed SNP markers, however, that does not mean that accuracy of genomic selection cannot be improved using high-density marker panels. The genomic matrix should evolve from a measure of realized additive relationships to an optimum measure of genetic similarity between individuals. The current method used to calculate the genomic relationship matrix gives the same weight to all the markers and thus does not guarantee the optimality of genetic similarity at QTL.

## Figures and Tables

**Table 1:** Variance components and heritability (SE) for different weighting scenarios of the prioritized 20K and the remaining 380K SNPs when the full panel (400K SNPs) was used to compute the genomic relationship matrix (average over 5 replicates).

Scenario ^2^	Weighting (%)		Genetic Variance	Residual Variance	Heritability
	20K ^1^	380K			
1 = (100,0)	100	0	0.196 (0.026)	0.671 (0.042)	0.228 (0.033)
2 = (90,10)	90	10	0.213 (0.018)	0.648 (0.032)	0.247 (0.023)
3 = (75,25)	75	25	0.232 (0.015)	0.633 (0.025)	0.268 (0.018)
4 = (50,50)	50	50	0.257 (0.016)	0.618 (0.021)	0.294 (0.018)
5 = (25,75)	258	75	0.279 (0.021)	0.619 (0.021)	0.311 (0.023)
6 = (PS ^3^,PS)	PS	PS	0.251 (0.032)	0.629 (0.037)	0.285 (0.037)
7 = Equal weights	Equal weights	Equal weights	0.247 (0.027)	0.692 (0.016)	0.263 (0.025)

^1^ Top 20K SNPs based on F_ST_ scores; ^2^ (x,y) are the percentages of the weights allocated to the prioritized top 20K and the remaining 380K SNPs, respectively; ^3^ contribution proportional to the SNP F_ST_ score.

**Table 2:** Distribution of off-diagonal elements (OD) of the genomic relationships matrix corresponding to the training and validation individuals using all 400 SNPs and for different weighting scenarios for the prioritized ^1^ (20K) and nonprioritized (380K) SNPs (in %).

OD	$\mathrm{Weights}\;(w_{i}\;\neq \;w_{j})$	$\mathrm{No}\;\mathrm{Weight}\;(w_{i}=1)$
OD < −0.05	2.32	1.61
0.05 < OD < −0.03	9.85	8.39
0.03 < OD < −0.01	28.18	29.35
−0.01 < OD < 0.01	33.48	36.14
0.01 < OD < 0.03	17.21	16.52
0.03 < OD < 0.05	5.52	4.86
OD > 0.05	3.46	4.86

^1^ SNPs selected based on F_ST_ scores.

**Table 3:** Distribution of off-diagonal elements (OD) of the genomic relationship matrix corresponding to the training and validation individuals using the prioritized ^1^ 20K SNPs and for different weighting scenarios (in %).

OD	Weights $\left(w_{i}\;\neq \;w_{j}\right)$	No Weight $\left(w_{i}=1\right)$	Scenario				
			(100,0) ^2^	(90,10)	(75,25)	(50,50)	(25,75)
OD < −0.05	0.92	0.00	2.32	1.66	0.92	0.25	0.03
−0.05 < OD < −0.03	4.60	0.77	9.85	8.72	6.81	3.67	1.42
−0.03 < OD < −0.01	30.26	29.77	28.18	29.16	30.45	31.72	31.22
−0.01 < OD < 0.01	43.93	52.43	33.49	35.54	38.86	44.57	49.81
0.01 < OD < 0.03	13.52	11.52	17.21	16.74	15.74	13.64	11.89
0.03 < OD < 0.05	4.04	3.30	5.52	5.01	4.36	3.68	3.36
OD > 0.05	2.73	2.21	3.46	3.19	2.86	2.49	2.27

^1^ SNPs selected based on F_ST_ scores; ^2^ (x,y) are the percentages of the weights allocated to the prioritized top 20K and the remaining 380K SNPs, respectively.

**Table 4:** Residual variance and log-likelihood of the model and the parameters (intercept and slope) of the regression of the estimated on the true breeding values for different weighting scenarios.

Scenario	Residual Variance	Intercept	Slope	−2LogL
1 = (100,0)	0.671 (0.04)	−1.208 (0.04)	0.664 (0.03)	26,409.13 (310.78)
2 = (90,10)	0.648 (0.03)	−1.228 (0.04)	0.675 (0.02)	26,397.40 (275.55)
3 = (75,25)	0.633 (0.03)	−1.244 (0.04)	0.683 (0.02)	26,404.90 (233.44)
4 = (50,50)	0.618 (0.02)	−1.240 (0.05)	0.682 (0.02)	26,489.84 (180.68)
5 = (25,75)	0.619 (0.02)	−1.185 (0.06)	0.651 (0.02)	26,746.99 (106.85)
6 = (PS,PS)	0.629 (0.04)	−1.205 (0.04)	0.662 (0.03)	26,619.76 (232.62)
7 = Equal weight	0.692 (0.02)	−0.921 (0.13)	0.505 (0.05)	27,378.30 (82.38)
